# Cumulative Adverse Childhood Experiences (ACEs) and School Outcomes During Adolescence: A Scoping Review

**DOI:** 10.1111/josh.70209

**Published:** 2026-08-10

**Authors:** Sarah A. Stoddard, Chelsea R. Moore, Carri S. Polick, Joshua M. Moss

**Affiliations:** ^1^ School of Nursing University of Michigan Ann Arbor Michigan USA; ^2^ University of Minnesota School of Medicine, Department of Pediatrics Division of General Pediatrics & Adolescent Health Minneapolis Minnesota USA; ^3^ ViaNova Healthcare Consulting & Research Firm Durham North Carolina USA; ^4^ School of Literature, Arts, and Sciences University of Michigan Ann Arbor Michigan USA; ^5^ Antitrust Division U.S. Department of Justice Washington DC USA

**Keywords:** adolescence, adverse childhood experiences, review, school

## Abstract

**Background:**

Emerging literature suggests cumulative adverse childhood experiences (ACEs), potentially traumatic experiences before age 18, are associated with poor school outcomes. This scoping review assessed the state of the literature on the association between cumulative ACEs and adolescent school outcomes.

**Methods:**

Following PRISMA‐ScR guidelines, four databases were searched. Two independent reviewers screened 7188 titles and abstracts, then 112 articles in full. Twenty‐four articles met eligibility criteria.

**Findings:**

Findings overwhelmingly supported a negative association between cumulative ACEs and school outcomes. Cumulative ACEs were associated with reduced likelihood of high school completion (*n* = 8), poor academic performance (*n* = 7), and school disengagement (*n* = 8). Nine studies identified mediational pathways between ACEs and school outcomes via biopsychosocial processes, mental/behavioral health, and school processes.

**Implications for School Health, Policy, Practice, and Equity:**

Findings underscore the importance of early identification strategies and preventive school health approaches that address trauma exposure to improve academic trajectories and reduce downstream educational disengagement.

**Conclusions:**

ACEs are negatively associated with school outcomes. Although additional research is needed, this review indicates that intervening on behavioral health and factors like school engagement, absenteeism, and academic achievement could result in improved school outcomes.

## 1 Introduction

More than 75% of students in the United States (US) report an Adverse Childhood Experience (ACE), or potentially traumatic experience before age 18 that includes maltreatment or significant household challenges [1]. Approximately 30% of children aged 12 have experienced two or more ACEs [2]. ACEs are associated with a range of negative behavioral, physical, and mental health outcomes across the life course, including early mortality [3, 4]. Emerging research indicates the accumulation of ACEs is also associated with poorer educational outcomes, like school disengagement and decreased academic achievement [5, 6, 7, 8, 9, 10]. However, this area of ACE research is nascent and expanding quickly. A synthesis of existing evidence regarding cumulative ACEs and adolescent school outcomes is needed.

### 1.1 Childhood Adversity

ACEs are substantial stressors that traditionally include abuse (physical, sexual, and emotional); neglect (physical and emotional); and household challenges, like parental separation/divorce, witnessing intimate partner violence, living with someone who struggles with mental illness or substance misuse, or living with someone who was incarcerated [4]. More recently, scholars have expanded the criteria to include additional adversities, like community violence, peer victimization, discrimination, foster care placement, and poverty [11, 12, 13]. Early research on childhood adversity primarily examined individual stressors [14], but in 1998 Felitti and colleagues advanced the field by introducing the concept of *cumulative* adversity, operationalized through an index of ACE types [4]. This cumulative approach is critical because it underscores the interrelatedness and co‐occurrence of ACEs [15, 16].

### 1.2 ACE Sequelae

The effect of ACEs can be explained through a biopsychosocial lens [17]. Childhood adversity is thought to exert profound and enduring effects on neurobiological development. Increased exposure to adversity is associated with persistently elevated stress hormones and structural and functional alterations in brain regions essential for executive functioning, learning, memory, and emotional regulation [18, 19, 20, 21]. Because the brain undergoes rapid growth from the neonatal period through early childhood and continues remodeling into late adolescence, it is highly susceptible to these disruptions, potentially compromising skills critical for academic success and healthy social relationships. Consistent with these mechanisms, higher ACE exposure is linked to poorer academic outcomes, including reduced achievement, lower engagement, increased absenteeism, and heightened risk of grade repetition or school dropout [5, 6, 7, 8, 22].

Lower educational attainment and ACEs are associated with similar negative, long‐term sequelae, including increased risk of chronic health conditions, poorer mental health [4, 8, 22, 23, 24, 25], health‐risk behaviors [3, 4, 8, 22, 23, 24], and earlier mortality [26, 27]. Educational attainment and the benefits it confers (e.g., health knowledge, cognitive skills, social status, labor market opportunities, income, and social support [27, 28]) may mediate the association between ACEs and various outcomes, presenting an opportunity for intervention. Given the neurocognitive sensitivity of adolescence and the long‐term health implications of both ACEs and educational attainment, clarifying the relationship between ACEs and educational outcomes is essential for effective prevention and intervention strategies.

### 1.3 Related Reviews of the Literature

Previous reviews have examined associations between individual childhood adversities and academic outcomes. Family violence exposure has been consistently linked to lower academic achievement [29]. Parental incarceration, particularly paternal incarceration, has been associated with poorer grades, grade retention, negative teacher evaluations, and reduced overall school success—findings regarding maternal incarceration were mixed [30]. Child maltreatment—including abuse and neglect—has been connected to special education involvement, absenteeism, grade retention, and poorer academic performance [31]. A meta‐analysis identified similar associations between various forms of violence exposure (e.g., sexual, emotional, physical, domestic, community, peer, and gang violence) and adverse academic outcomes, including school dropout, grade repetition, and participation in remedial classes [32]. While these reviews underscore the influence of specific adversities on educational outcomes, ACEs often co‐occur, thus there is a need to assess the impact of cumulative adversity on school outcomes.

To date, we have found only one review that examined the impact of cumulative ACEs on academic outcomes. This systematic review and meta‐analysis [9] found a modest negative correlation between ACE scores and objective measures of academic achievement (e.g., grades and standardized test scores). However, the analysis excluded other important school‐related outcomes (e.g., school connectedness, future orientation). It also combined data across childhood and adolescence, limiting insight into adolescence—a critical developmental stage marked by rapid socioemotional changes and proximity to key academic milestones, like high school graduation. The current review addresses these limitations, guided by two questions: (1) What is the effect of cumulative childhood adversity on adolescent school outcomes? (2) What gaps remain in the literature regarding ACEs and adolescent educational experiences?

## 2 Methods

This scoping review followed the Preferred Reporting Items for Systematic Reviews and Meta‐Analysis extension for Scoping Reviews (PRISMA‐ScR) guidelines [33]. While this is a scoping review—aimed at broadly assessing the state of the literature and identifying knowledge gaps—we followed the updated guidance that supports the use of methodological processes that increase rigor, reproducibility, and transparency (e.g., a systematized search with two independent reviewers). Our protocol provides additional methodologic details [34].

### 2.1 Search Strategy

We searched four databases (PubMed, PsycINFO, Scopus, and Educational Abstracts) in December 2022, guided by an experienced informationist. We used over 70 search terms with Boolean logic and Medical Subject Headings. Examples include “Adverse Childhood Experiences,” “Adolescents,” “Educational attainment,” and “School success.” The protocol includes an example search string [34]. We conducted a second search in April 2026—using the same search strategy with December 2022 set as the start date—to identify more‐recent articles.

### 2.2 Screening and Selection

**FIGURE 1:**
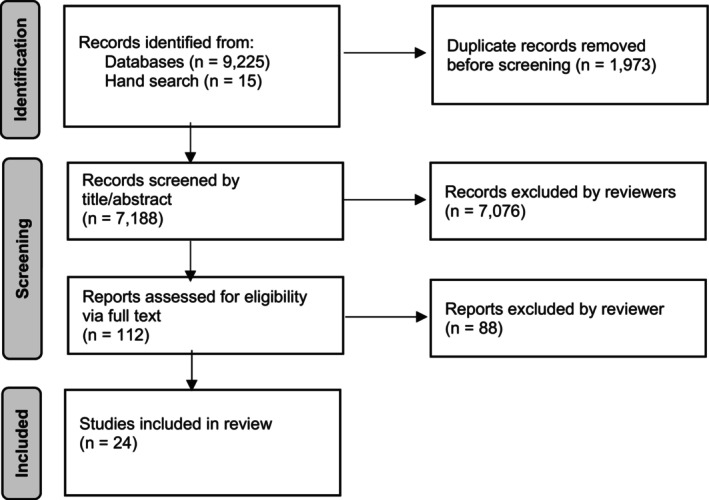
PRISMA [33] flow diagram of article identification, selection, and inclusion. Duplicate records removed before screening (*n* = 1973).

Records from all databases were imported into EndNoteX9 to delete duplicates, then imported into Rayyan.ai for screening. Two reviewers screened titles and abstracts independently then compared results. A second round of full‐text screening was then conducted (Figure 1). A third reviewer audited approximately 100 records throughout the screening process to ensure fidelity to inclusion and exclusion criteria. A fourth reviewer was available to discuss discrepancies. Inter‐rater reliability was 99%.

### 2.3 Inclusion and Exclusion Criteria

We sought empirical articles reporting on ACEs and adolescent school outcomes in the United States. Articles must have been original, quantitative research using a US‐based sample, published in English in peer‐reviewed journals after 1995. We included longitudinal and cross‐sectional designs utilizing primary or secondary data analysis. Studies included national, regional, and specific population subgroups. The 1995 cutoff corresponds with the start of the original ACE study [4]. We *excluded* reviews, policy briefs, narrative papers, gray literature (e.g., dissertations, conference papers), and studies published in non‐English languages.

Specifically, studies had to *include* the cumulative assessment of three or more ACEs, including: physical, emotional, or sexual abuse; neglect; witnessing domestic violence; parental separation/divorce; living with someone with mental illness, substance misuse, or who has been imprisoned; parental or sibling death; witnessing community violence; poverty; or discrimination. Studies that assessed only 1–2 ACEs or used vague terms, like “maltreatment,” were *excluded*.

Studies had to *include* school outcomes between ages 12 and 18 years, such as academic achievement (e.g., grades, graduation) or factors associated with school disengagement (e.g., absenteeism). Studies were *excluded* if they only assessed school outcomes for children younger than 12 years old or only examined school outcomes as a mediator of other outcomes without providing information on the ACEs‐to‐school outcomes path.

### 2.4 Data Extraction

A detailed extraction tool was used to compare the content of eligible studies, including sample characteristics, study design, and key findings. Extracted data from each article was reviewed by at least two researchers to ensure accuracy and completeness.

## 3 Findings

### 3.1 Study and Sample Characteristics

Twenty‐four studies met all inclusion criteria (Table 1, Figure 1). Included studies were published between 2016 and 2026 (March). A majority were secondary data analyses (*n* = 21) and cross‐sectional studies (*n* = 13). Fifteen studies were nationally representative [22, 35, 36, 37, 38, 39, 40, 41, 42, 43, 44, 45, 46, 47, 48]: eight representative of the general population [22, 35, 36, 37, 42, 45, 47, 48], one of children born in large US cities [46], two of children with autism [38, 39], and four of US families with Child Protective Services [CPS] contact or deemed “high risk” for child maltreatment [40, 41, 43, 44]. Five studies focused on state or regional level data [5, 49, 50, 51, 52].

**TABLE 1:** Key findings.

First author, year	Data source	Sample size & geographic area	Sample characteristics	Key findings
Cross‐sectional studies (*n* = 13)				
Augustinović, 2026	National Survey of Children's Health (NSCH) 2018–2019	*n* = 31,006 Nationally representative	Age: 10–17 years (31% 10–12 years) Female: 48% Race/ethnicity: 70% NH White, 7% NH Black, 12% Hispanic, 5% Asian, 12% Native American/Hawaiian, 1% multiracial/other ACE Prevalence: 1+ ACEs: 43% 4+ ACEs: 5%	Greater exposure to ACEs was associated with higher odds of school disengagement. Income (categorized as below poverty, low income, middle income or high income) moderated this association. While low‐income youth experienced the lowest school engagement overall, high‐income youth experienced the greatest decline in school engagement as their ACE count increased.
Boccio, 2025	Florida Youth Substance Abuse Survey 2022	*n* = 19,857 Florida	Age: 13–19 years (avg: 15.88) Female: 52% Race/ethnicity: 53% NH White, 14% NH Black, 26% Hispanic ACE Prevalence: 1+ ACEs: 68% 4+ ACEs: 24%	Cumulative ACEs were associated with greater odds of truancy and the frequency of days skipped in a dose–response manner. The association between ACEs and odds of truancy is partially mediated by school attachment (strongest mediator), school grades, and school suspension. The association between ACEs and number of days skipped is partially mediated by school grades (strongest mediator), school attachment, school suspension and parental supervision (weakest mediator).
Crouch, 2019	NSCH 2016	*n* = 31,707 Nationally representative	Age: 6–17 years (58% 6–12 years) Female: 49% Race/ethnicity: 53% NH White, 12% NH Black, 10% NH Other, 25% Hispanic ACE Prevalence: 1+ ACEs: 51% 4+ ACEs: 8%	Cumulative ACEs were associated with greater odds of school disengagement, school absenteeism, and repeating a grade.
Duke, 2020	Minnesota State Survey 2016	*n* = 81,885 Minnesota	Age: 13–19 years Grade: 9th (55%) & 11th (45%) graders; 13–19 years old (avg: 15.5) Female: 50% Race/ethnicity: 71% NH White, 9% Hispanic, 7% Multiple races (NH), 6% NH Asian American, 6% NH Black, 1% NH American Indian, < 1% NH Pacific Islander ACEs Prevalence: 1+ ACEs: 36% 4+ ACEs: 5%	Cumulative ACEs were associated with increased odds of having no plans to graduate, cutting 3+ days of school within the past month, and having below average academic achievement. The association between ACEs and absenteeism was marginally attenuated (moderated) by school connection.
Ghaffari, 2024	NSCH 2022	*n* = 24,173 Nationally representative	Age: 10–17 years (44% 10–13 years) Female: 48% Race/ethnicity: 76% NH White, 8% NH Black, 6% Asian, 2% AI/AN/NH/Other Pacific, 8% Multiple Races ACEs Prevalence: 1+ ACEs: 40% 3+ ACEs: 9%	Children with more ACEs (vs. children with no ACEs) had lower odds of receiving high‐performance grades (mostly As or mostly As and Bs) than middle and low‐performance grades (ranging from mostly Bs to Ds and lower).
Gussin, 2025	NSCH 2016–2021	*n* = 4997 Nationally representative of youth with autism spectrum disorder	Age: 6–17 years (avg: 11.72) Female: 21% Race/ethnicity: 50% NH White, 28% Hispanic/Latino, 14% NH Black, 8% other ACE Prevalence: Avg cumulative score: 2.0 (95% CI: 1.9–2.2)	Cumulative ACEs were associated with lower odds of school attendance, grade progression, and school engagement, as well as a lower scores on the school success index (that accounted for the three outcomes together). When depression and anxiety symptoms were included in the analysis, the associations between ACEs and the school outcomes were attenuated, suggesting potential mediation effects. The ACEs‐attendance association became non‐significant. The associations between ACEs and grade progression, school engagement and the school success index were weakened but remained significant.
Hartwell, 2024	NSCH 2016–2021	*n* = 4717 Nationally representative of youth with autism spectrum disorder	Age: 6–17 years (39% 6–10 years, 36% 11–14 years) Female: Not reported Race/ethnicity: 50% NH White, 13% NH Black, < 1% Indigenous, 3% Asian, 5% Multiracial/other, 28% Hispanic ACE Prevalence: 1+ ACEs: 62% 4+ ACEs: 12%	Cumulative ACEs were directly associated with poorer school outcomes (a latent variable indicated by: caring about doing well in school, completing homework, and reported problems at school). ACEs were indirectly associated with poorer school outcomes through an inverse relationship with support services—when support services were increased, school outcomes improved.
Kernan, 2025	Florida Youth and Substance Abuse Survey 2022	*n* = 23,078 Florida	Grades: 9th–12th Female: 51% Race/Ethnicity: 47% NH White, 14% Black, 18% Latinx, 3% Asian, 1% Native American, 18% Multiracial/other ACEs Prevalence: 1+ ACEs: 69% 4+ ACEs: 24%	Cumulative ACEs were associated with greater odds of school suspension.
Johnson R., 2021	Maryland Youth Risk Behavior Survey/Youth Tobacco Survey (YRBS/YTS) 2018	*n* = 40,188 Maryland	Grades: 9th (28%), 10th (26%), 11th (23%) and 12th (23%) Female: 49% Race/ethnicity: 53% White, 20% Black, 12% Latinx, 4% Asian, 7% Multiracial, 4% Other race/ethnicity ACE Prevalence: 1+ ACEs: 56% 3+ ACEs: 15%	Cumulative ACEs were associated with increased prevalence of poor school performance and fighting at school.
Lurie, 2023	Primary data collection from 2 longitudinal studies of community‐based samples	*n* = 408 Seattle, Washington	Combined sample avg. age: 12.7 years (SD: 2.3) Subset 1 (*n* = 192): 10–18 years old (avg: 14.1, SD: 2.8) Subset 2 (*n* = 216): 10–13 years old (avg: 11.5, SD: 0.5) Female: 46% Race/ethnicity: 53% White, 19% Black, 11% Latino, 10% Asian, 7% other ACE Prevalence: Avg threat score: 0.9 (SD: 2.0) Avg deprivation score: 0.7 (SD: 0.9)	Cumulative experiences of threat and deprivation were each independently associated with worse academic performance, even after controlling for the alternative form of adversity. Experiences of threat and deprivation were negatively associated with growth mindset of intelligence when examined independently. When examined simultaneously, the association between threat and growth mindset was largely unchanged, but the association between deprivation and growth mindset became non‐significant. The association between threat and academic performance was partially mediated by growth mindset.
Moore, 2024	Panel Study of Income Dynamics (PSID)	*n* = 8009 Nationally representative	Age: 18–91 years (avg: 46.3, SD: 15.9) Female: 57.5% Race/ethnicity: 62% NH White, 5% Hispanic, 27% NH Black, 6% NH Other/Multi ACE Prevalence: 1+ ACEs: 49% 4+ ACEs: 4%	Cumulative ACEs were directly associated with HS dropout. Depression and substance use partially mediated this association, but not anxiety.
Suleiman, 2021	NSCH 2016–2018	*n* = 41,648 Nationally representative	Age: 12–17 years (avg: 14.5) Female: 49% Race/ethnicity: 51% NH White, 25% Hispanic, 15% NH Black, 10% NH other ACE Prevalence: 1+ ACEs: 53% 4+ ACEs: 8%	Cumulative ACEs were associated with an increased number of school engagement/performance problems (using an index that captured disengagement, absenteeism, problems at school requiring calls to caregivers and grade repetition). This association was partially mediated by behavioral health conditions (including anxiety, depression, and attention deficit hyperactivity disorder or conduct problems).
Tsevat, 2025	National Health Interview Survey 2021–2022	*n* = 10,794 Nationally representative	Age: 6–17 years (avg: 11.60) Female: 49% Race/ethnicity: 51% NH White, 26% Hispanic, 12% NH Black, 6% other, 5% NH Asian ACE Prevalence: 1+ ACEs: 24% 4+ ACEs: 3%	Cumulative ACEs were associated with health‐related absenteeism and health‐related chronic absenteeism, in a dose–response manner. General health status partially mediated these associations.
Longitudinal studies (*n* = 11)				
Brumley, 2021	Primary data from 3 primary care clinics associated with a large pediatric hospital	*n* = 99 Mid‐Atlantic region	Age: 13–16 years (avg: 15.2) Female: 50% Race/ethnicity: 87% NH Black, 3% NH White, 3% NH Asian, 2% Hispanic/Latino, 4% Other race/ethnicity ACE Prevalence: 1+ ACEs: 75% 4+ ACEs: 17%	ACE exposure was not significantly associated with the number of academic goals reported, achievability appraisals, or support appraisals, after accounting for potential confounders, including externalizing behaviors. A near‐significant association was found between youth ACE exposure and feeling less supported in their academic goals.
Giovanelli, 2016	Chicago Longitudinal Study (CLS)	*n* = 1202 Chicago, Illinois	Age: 22–24 years Female: 54% Race/ethnicity: 93% NH Black, 7% Hispanic ACE Prevalence: 1+ ACEs: 62% 4+ ACEs: 13%	Cumulative ACEs were associated with reduced odds of HS graduation.
Giovanelli, 2020	CLS	*n* = 1341 Chicago, Illinois	Age: 22–24 (75%) or 35–37 years Female: 53% Race/ethnicity: 93% NH Black, 7% Hispanic ACE Prevalence: 1+ ACEs: 70% 4+ ACEs: 19%	Cumulative ACEs were negatively associated with graduating HS. This association was consistently found in subgroup analyses comparing males versus females, and youth living in more versus less impoverished areas.
Kallman, 2025	Longitudinal Studies on Child Abuse and Neglect (LONGSCAN)	*n* = 494 Nationally representative of families with CPS contact	Baseline age: 4 years; 16 years at final interview (5 timepoints) Female: 46% Race/ethnicity: 46% Black, 40% White, 5% Mixed/other race/ethnicity, 7% Hispanic/Latino; 1% Native American, 1% Asian ACE Prevalence: Avg ACE score: 5.10 (SD: 1.90)	While cumulative ACEs were not directly associated with reading score, ACEs were indirectly associated with lower reading scores through hyperactivity/impulsivity symptoms (not inattention symptoms).
Johnson M., 2018	Primary data from juveniles in the Florida Department of Juvenile Justice system	*n* = 2558 Florida	Age: 12–16 years (avg: 14) Female: 18% Race/ethnicity: 58% NH Black, 31% NH White, 10% Latino/a, < 1% Other race ACE Prevalence: 1+ ACEs: 97% Avg ACE score: 3.1 (SD: 1.8)	Cumulative ACEs were associated with lower expectations of graduating HS in a dose–response manner.
Leban, 2021	LONGSCAN	*n* = 490 Nationally representative of families with CPS contact	Baseline age: 4–6 years; 18 years at final interview (6 timepoints) Female: 58% Race/ethnicity: 62% Black, 38% White ACE Prevalence: 1+ ACEs: Not reported. 4+ ACEs: 61%	Cumulative ACEs were positively associated with odds of school dropout for Black and White students after accounting for school suspension (by 12 years of age) and demographic covariates. An interaction was identified between school suspension and ACEs regarding school dropout for Black youth; the impact of school suspension on HS dropout was significantly stronger for Black youth who experienced more ACEs.
Lui, 2023	National Longitudinal Survey of Youth 1979 cohort and the children/young adult cohort	*n* = 5521 Nationally representative	Baseline age: 0–4 years; outcomes surveyed at 15–18 years (4 timepoints) Female: 49% Race/ethnicity: 23% NH Black, 51% NH White, 20% Hispanic/Latinx, 6% other ACE Prevalence: 1+ ACEs: 70% 4 ACEs: 4%	Early childhood ACEs (at ages 0–4) were directly associated with HS dropout. Mediation analyses indicated multiple indirect pathways from early childhood ACEs to high school dropout via behavioral problems and low academic performance at ages 6–13 and cannabis use at ages 15–18.
Morrow, 2018	LONGSCAN	*n* = 728 Nationally representative of families with CPS contact	Baseline age: 4–6 years old; 18 years at final interview (6 timepoints) Female: 56% Race/ethnicity: 54% Black, 26% White, 14% Mixed/other race/ethnicity, 6% Hispanic/Latino ACE Prevalence: Avg ACE score: 3.6 (SD: 2.1)	Cumulative ACEs were associated with lower reading scores (at age 16) and greater odds of school dropout (at age 18). The association between ACEs and school dropout was partially mediated by reading scores, externalizing problems, and internalizing problems (at age 16).
Mullins, 2021	National Study of Child and Adolescent Wellbeing II (NSCAW II)	*n* = 583 Nationally representative of families involved with CPS	Age: 11–15 years, then 12–16 years Female: 56% Race/ethnicity: 50% White, 27% Black 26% Hispanic, 14% American Indian, 12% other race/ethnicity, 6% Asian/Hawaiian/Pacific Islander ACE Prevalence: 1+ maltreatment ACEs substantiated: 53% Avg maltreatment score: 2.3 (SD: 1.9)	There was a significant total effect of cumulative maltreatment on math scores, but after holding other variables constant (including trauma symptoms), the direct effect became non‐significant. Cumulative maltreatment was not associated with academic engagement or reading scores.
Pan, 2020	National Longitudinal Study of Adolescent Health (Add Health), waves 3 (2001–2002) and 4 (2009)	*n* = 14,823 Nationally representative	Age at Wave 3: 18–26 years (avg: 22.0) Female: 53% Race/ethnicity: 63% White, 21% Black, 7% Asian, 4% Multiracial, 3% Native American ACE Prevalence: 1+ ACEs: 68% 3+ ACEs: 19%	Cumulative ACEs were associated with reduced odds of graduating HS.
Pierce, 2022	Future of Families and Child Wellbeing Study (FFCWS)	*n* = 3382 Nationally representative of children born in large, urban areas	Avg age at final survey: 15.2 years Female: 51% Race/ethnicity: 37% White, 28% Hispanic, 25% Black, 10% other race/ethnicity ACE Prevalence: 1+ ACEs: 81% 4+ ACEs: 21%	Cumulative ACEs at ages 0–5 and at age 9 were each associated with increased odds of school suspension/expulsion at age 15. No interactions were found between cumulative ACEs and several demographic characteristics (including race/ethnicity, social class, gender, or ADHD diagnosis) regarding odds of school suspension/expulsion.

Abbreviations: aOR, adjusted odds ratio; FFCWS, fragile families and child wellbeing study; HH, household; HS, high school; NH, non‐Hispanic; PR, prevalence ratio.

Sample sizes ranged from 99 [53] to 81,885 [5] participants. Most studies had a near‐even sex distribution; two studies predominantly reported on male participants [38, 50]. Nine studies included a large proportion of minoritized participants (e.g., non‐White or low‐income) [6, 40, 41, 43, 44, 46, 50, 53, 54]. Across studies that reported ACE prevalence, 24%–97% of participants experienced at least one ACE and 3%–61% experienced four or more ACEs.

### 3.2 ACE Measurement

The most comprehensive studies assessed 14 types of ACEs [54], followed by 12 [44], and 10 types [5, 38, 40, 41, 50, 51, 52, 53] (Table 2). Most studies employed a composite ACE measure developed in alignment with the original ACE questionnaire (i.e., items focused on child maltreatment and household dysfunction). Fifteen studies included measures with expanded ACEs (e.g., neighborhood violence, discrimination, food insecurity, or foster care placement) [5, 6, 35, 36, 37, 38, 39, 40, 42, 45, 47, 48, 49, 53, 54]. Three studies supplemented parent‐/youth‐reported ACE information with official CPS records [40, 41, 43].

**TABLE 2:** Specific adverse childhood experiences (ACEs) and school outcomes assessed in included studies.

First author, year	ACEs	School outcomes
Cross‐sectional studies (*n* = 13)		
Augustinović, 2026	caregiver divorce/separation caregiver died caregiver incarceration witness HH violence neighborhood violence HH mental illness HH substance misuse racial/ethnic discrimination	School engagement Age measured: 10–17 years
Boccio 2025	emotional abuse physical abuse sexual abuse divorce domestic abuse HH substance misuse HH mental illness HH incarceration emotional neglect physical neglect	Truancy (yes/no and number of days skipped) Average school grades School attachment Feel safe in school Age measured: 13–19 years
Crouch, 2019	HH mental illness HH substance abuse parental separation/divorce HH incarceration parental death witness neighborhood violence witness HH violence economic hardship racial/ethnic mistreatment	Lack of school engagement School absenteeism Grade repetition Age measured: 6–17 years
Duke, 2020	physical abuse sexual abuse by family member sexual abuse by non‐family member HH alcohol abuse HH substance abuse (illegal/prescription) caregiver incarceration verbal/emotional abuse witnessing HH violence food insecurity housing instability	No plan to graduate Past month unexcused absences Low academic achievement School connection (as moderator) Age measured: 13–19 years
Ghaffari, 2024	caregiver divorce/separation caregiver died caregiver incarceration witnessing HH domestic violence neighborhood violence HH mental illness HH substance misuse	Grades in school Age measured: 10–17 years
Gussin 2025	Material hardship Caregiver divorce/separation Caregiver died Caregiver incarcerated Witness HH violence Neighborhood violence HH mental illness HH substance misuse Racial/ethnic discrimination Bullied	School engagement Grade progression School attendance School Success (sum of engagement, progression, and attendance) Age measured: 6–17 years
Hartwell, 2024	Material hardship Caregiver divorce/separation Caregiver died Caregiver incarcerated Witness HH violence Neighborhood violence HH mental illness HH substance misuse Racial/ethnic discrimination	Latent school outcomes variable, indicated by: – Caring about doing well in school – Completing homework – Reported problems at school Age measured: 6–17 years
Johnson R., 2021	HH mental illness HH substance abuse or gambling HH criminal justice involvement caregiver verbal abuse food insecurity	Poor school performance (grades) Physical fighting at school Age measured: 9‐12th grade
Kernan, 2025	physical abuse, sexual abuse, emotional abuse, physical neglect, emotional neglect HH mental illness, HH incarceration, HH violence, parental divorce/separation HH substance misuse	School suspension
Lurie, 2023	Threat: physical abuse sexual abuse emotional abuse HH violence other interpersonal violence Deprivation: physical neglect emotional neglect cognitive deprivation material deprivation	Growth mindset of intelligence Academic performance (including grades for 4 academic subjects; grade repetition; remedial class participation; other academic‐related problems)
Moore, 2025	emotional abuse physical abuse emotional neglect witnessing HH violence parental substance misuse parental mental illness parental separation/divorce	High school dropout
Suleiman, 2021	HH mental illness, HH substance abuse, caregiver divorce/separation, caregiver incarceration, caregiver death, neighborhood violence domestic violence, economic hardship, social discrimination	Index of school engagement and performance including: Motivation to do well in class Completing required homework Missed school 11+ days Grade repetition (after kindergarten) 2+ times that school contacted caregivers to discuss any problems Age measured: 12–17 years
Tsevat, 2025	caregiver incarcerated HH mental illness HH substance misuse verbal abuse neglect racial/ethnic discrimination neighborhood violence	Health‐related school absenteeism Health‐related chronic absenteeism
Longitudinal studies (*n* = 11)		
Brumley, 2021	physical abuse sexual abuse HH mental illness caregiver substance use having a loved one in prison verbal abuse emotional abuse physical neglect domestic violence neighborhood dysfunction [measured separately with the 20‐item Neighborhood Climate Scale]	Academic goal setting (number of academic goals reported and if goals were future oriented) Appraisals of goals (perceived achievability & support) Age measured: 13–16 years old
Giovanelli, 2016	physical abuse sexual abuse parental substance abuse long‐term parent absence/separated or divorced parents death of parent/sibling/close friend/relative neglect being a victim of a violent crime witnessing shooting or stabbing frequent family conflict Age measured: 0–18 years	HS graduation by age 21 (administrative records and self‐reports)
Giovanelli, 2020	Physical abuse Child welfare reports (abuse or neglect) Sexual abuse Neglect Foster care/out of home placement Parental substance abuse prolonged absence of a parent/divorce death of a parent death of a sibling death of a close friend witnessing a violent crime being a victim of a violent crime frequent family conflict family financial problems	HS graduation by age 25 (school records and self‐report)
Kallman, 2025	physical abuse sexual abuse emotional abuse neglect witnessed HH violence HH substance misuse HH incarceration caregiver mental illness parental divorce/separation caregiver died Occurred between ages 0 and 14 (caregivers and/or child report)	Academic achievement (based on standardized reading score) Age measured: 16 years
Johnson M., 2018	physical abuse sexual abuse HH mental illness HH substance abuse parental separation HH member incarcerated emotional abuse emotional neglect physical neglect witnessing domestic violence	Expect to graduate from HS Age measured: 12–16 years
Leban, 2021	physical abuse caregiver perpetration of sexual abuse emotional abuse physical neglect (failure to provide) parental mental illness parent criminality lack of supervision parent intimate partner violence family trauma parent substance abuse Assessed as occurring between age 0 and 12 years	School dropout School suspension Age measured: 15–18 years
Lui 2023	maternal substance misuse HH poverty emotional neglect cognitive deprivation	High School completion
Morrow, 2018	physical abuse sexual abuse emotional abuse neglect caregiver mental illness HH substance use HH incarceration witnessed family violence Assessed as occurring between age 0 and 14 years	School dropout (assessed at age 18) Reading achievement (assessed at age 16)
Mullins, 2021	physical abuse, sexual abuse substance‐abusing parent emotional abuse failure to provide lack of supervision abandonment domestic violence educational maltreatment exploitation substance exposure other type of abuse	Academic engagement (11 items from the Drug Free Schools Outcome Study; at 12–16 years) Math and reading achievement (22 item Woodcock‐ Johnson III Tests of Achievement; at 14–19 years)
Pan, 2020	physical abuse sexual abuse perpetrated by a parent or other adult caregiver emotional neglect physical neglect foster care placement homelessness stopped or detained by police	HS graduation Assessed in wave 4 survey (˜24–32 years old)
Pierce, 2022	physical abuse HH depression/anxiety HH substance abuse parental incarceration emotional abuse emotional neglect physical neglect intimate partner violence Age measured: 1, 3, 5, and 9	Suspended or expelled in the last 2 years Age measured: 15 years

Abbreviations: HH, household; HS, high school.

### 3.3 School Outcomes Measurement

Most studies examined multiple school outcomes while six studies only assessed high school graduation [6, 45, 46, 50, 54, 55] (Tables 2 and S1). Eight studies assessed high school graduation or dropout [6, 22, 41, 42, 43, 45, 50, 54]. Proximal outcomes included academic performance/achievement (*n* = 8) [5, 37, 40, 43, 44, 49, 51, 56], school engagement/connectedness (*n* = 8) [5, 35, 36, 38, 39, 44, 47, 51], absenteeism (*n* = 6) [5, 36, 38, 47, 48, 51], school suspension/expulsion (*n* = 3) [41, 46, 52], grade repetition (*n* = 3) [36, 38, 47], problems at school reported to caregivers (*n* = 2) [39, 47], academic goal setting (*n* = 1) [53], fighting at school (*n* = 1) [49], growth mindset of intelligence (*n* = 1) [56], and perceived safety at school (*n* = 1) [51].

### 3.4 Associations Between ACEs and School Outcomes

A vast majority of studies (*n* = 22) found a significant association between cumulative ACEs and poorer school outcomes. We report key findings by type of study design with greater detail provided in Table 1.

#### 3.4.1 Nationally Representative, Longitudinal Evidence

Seven articles (29%) used longitudinal data from nationally representative samples, providing robust evidence of the association between increased ACEs and poorer school outcomes [40, 41, 42, 43, 44, 45, 46]. Notably, only two studies used data representative of the *general* population at a national level (i.e., National Longitudinal Study of Adolescent Health [AddHealth] [45] and National Longitudinal Survey of Youth [42]). Both studies found a negative association between cumulative ACEs and odds of graduating high school [42, 45], with Pan and colleagues [45] reporting each additional ACE type experienced was associated with 25% lower odds of high school graduation. The next most generalizable study analyzed data collected as part of the Future of Families and Child Wellbeing Study [46] and reported that youth with four or more ACEs (compared to those with no ACEs) had significantly increased odds of experiencing school suspension or expulsion [46].

Four longitudinal studies were nationally representative of families with previous CPS contact or who were deemed “high risk” for child maltreatment. One study used data from the National Survey of Child and Adolescent Wellbeing (NSCAW II) [44] and three studies used the Longitudinal Studies of Child Abuse and Neglect (LONGSCAN) data [40, 41, 43]. The three LONGSCAN studies reported associations between cumulative ACEs and poorer school outcomes, including increased odds of high school dropout [41, 43, 45] and reduced reading achievement [40, 43]. Alternatively, the study using NSCAW II data found no significant associations between ACEs and school outcomes, including school engagement, reading or math achievement, after controlling for trauma symptoms and other factors [44]. Importantly, this study treated ACEs and trauma symptoms as separate, uncorrelated independent variables in a path analysis, but bivariate analyses revealed a significant correlation between the two (*r* = 0.23).

#### 3.4.2 Other Longitudinal Evidence

Four studies used a longitudinal study design with regionally‐based samples [6, 50, 53, 54]. Of note, all four studies used samples that included a higher proportion of non‐Hispanic Black participants and reported higher‐than‐expected ACE scores (compared to national estimates) [6, 50, 53, 54]; three reported samples predominantly residing in low‐income neighborhoods [6, 53, 54]. Three studies found a significant association between cumulative ACEs and lower odds of high school graduation; this was the only outcome examined in all three studies [6, 50, 54]. The fourth study examined academic goal setting and perceptions of goal achievability and support [53]. No significant associations were found between ACE scores and these outcomes, although the negative association with perceived support approached significance (*p* = 0.06). It is noteworthy that this latter analysis accounted for externalizing behaviors which were significantly correlated with ACE scores (*r* = 0.44) and significantly associated with the total number of academic goals. Moreover, this study employed the smallest sample size (*n* = 99) of all included studies [53].

#### 3.4.3 Nationally Representative, Cross‐Sectional Evidence

Eight studies used cross‐sectional designs and large, nationally representative samples [22, 35, 36, 37, 38, 39, 47, 48]. Data were from the Panel Study of Income Dynamics (PSID) [22], the National Health Interview Survey (NHIS) [48], and the National Survey of Children's Health (NSCH) [35, 36, 37, 38, 39, 47]. The NSCH is a study of the general population under age 18; however, two studies focused on sub‐samples of youth with autism spectrum disorder [38, 39]. Altogether, the eight studies found associations between cumulative ACEs and negative school outcomes, including low school engagement [35, 36, 38, 39, 47], absenteeism [36, 38, 47, 48], grade repetition [36, 38, 47], lower grades [37], high school dropout [22], and having caregivers contacted multiple times regarding problems at school [39, 47].

#### 3.4.4 Other Cross‐Sectional Evidence

Five studies used cross‐sectional designs with regionally drawn samples [5, 49, 51, 52, 56], including representative samples of public school students from Florida [51, 52], Minnesota [5], and Maryland [49], as well as a community‐based sample from Seattle, Washington [56]. All studies found associations between cumulative ACEs and poorer school outcomes, including poorer school performance [5, 49, 56], absenteeism [5, 51], physically fighting at school [49], lower odds of having plans to graduate from high school [5], school suspension [52], and lower growth mindset [56].

#### 3.4.5 Mediators and Moderators

**FIGURE 2:**
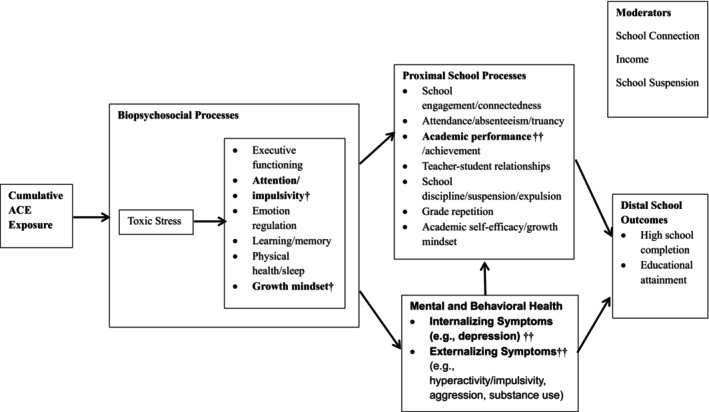
Pathways linking cumulative ACE exposure to adolescent school outcomes. Cumulative ACE exposure may influence school outcomes through biopsychosocial, mental/behavioral health, and more proximal school processes. Note. ^†^mediator between ACEs and proximal school processes; ^††^mediator between ACEs and high school completion.

Several studies examined the association between cumulative ACEs and school outcomes using mediation or moderation analyses (see Figure 2). Eleven studies assessed for mediating factors [22, 38, 39, 40, 42, 43, 44, 47, 48, 51, 56] with five of those published within the past two years [22, 38, 39, 48, 51] and three using longitudinal data [40, 42, 43]. Nine studies identified significant mediators [22, 39, 40, 42, 43, 47, 48, 51, 56] and one identified potential mediators (without using formal mediation analysis) [38].

Three studies investigated the pathway from ACEs to high school dropout [22, 42, 43], identifying the following partial mediators: behavioral problems (including internalizing and externalizing symptoms) [22, 42, 43] and decreased academic performance [42, 43]. However, findings suggest not all behavioral problems mediate the ACEs‐high school dropout association similarly. For example, one study found externalizing problems (e.g., aggression, substance use) were associated with increased odds of dropout, but internalizing problems (e.g., depression, anxiety) were associated with decreased odds of dropout [43]. Findings from another study build upon this finding. When looking specifically at depression, anxiety, and substance use as individual mediators, researchers found depression and substance use were associated with increased odds of dropout, but anxiety was not associated with dropout [22]. This suggests distinct behavioral problems may uniquely mediate the link between ACEs and high school dropout.

Two studies investigated mediators within the association between ACEs and academic achievement [40, 56]. One study reported hyperactivity/impulsivity symptoms completely mediated the association between ACEs and lower reading scores [40], and another study reported a lower growth mindset regarding intelligence partially mediated the association between threat‐based ACEs and overall academic performance [56].

When investigating the association between ACEs and absenteeism, one study found school attachment, grades, and history of suspension were partial mediators [51], and another study identified general health status as a partial mediator [48]. The study that did not conduct formal mediation analysis suggested depression and anxiety symptoms may be complete mediators of the association between ACEs and school attendance because they attenuated ACE effects on attendance to non‐significant levels when included in a single model [38].

Two studies investigated mediators within the association between ACEs and measures that capture a combination of many negative school outcomes [39, 47]. One study found behavioral health conditions (encompassing anxiety, depression, attention deficit hyperactivity disorder, and conduct problems) were a partial mediator [47]. One study, focused on youth with autism spectrum disorder, found that the level of support services received was a partial mediator [39, 47]. Lastly, one study (focused on youth with autism spectrum disorder) did not conduct formal mediation analysis, but suggested potential mediation effects to be explored in future research based on results from serial regression models [38]. Their findings suggested depression and anxiety symptoms may completely mediate the association between ACEs and absenteeism, and may partially mediate the association between ACEs and grade repetition, school disengagement and a combination measure of all three previous factors [38].

Of note, one study investigated whether academic engagement mediated the association between maltreatment‐specific ACEs and low math/reading achievement, but found no significant effects (total/direct or indirect) [44]. However, trauma symptoms were included in the full path analysis and maltreatment was significantly correlated with trauma symptoms [44].

Five studies assessed for factors that may moderate ACEs‐to‐school outcome associations [5, 6, 35, 41, 46]. One study reported school connection marginally buffered some associations between ACEs and absenteeism or low academic achievement [5]. This attenuating effect was not found for the association between ACEs and expectations to graduate high school. Another study investigated for interaction effects between cumulative ACEs and school suspension regarding school dropout, but found only a significant interaction for Black students [41]. The authors report that the association between school suspension and dropout was significantly stronger for Black youth who had experienced cumulative ACEs [41]. A third study found that income moderated the association between ACEs and school disengagement [35]. While they found youth from low‐income households (the second lowest category) experienced the greatest disengagement across all ACE levels, youth with the highest household incomes experienced the sharpest decline in school engagement as ACE exposures increased [35].

The two remaining studies that assessed for moderating effects found no significant interactions. One study investigated various possible interactions between ACEs and demographic variables (including race/ethnicity, social class, gender, and attention deficit hyperactivity disorder diagnosis) in association with school suspension/expulsion; no significant effects were found [46]. The second study assessed whether participation in a specific preschool intervention buffered the negative association between ACEs and high school graduation, but found no significant interaction [6].

## 4 Discussion

This scoping review highlights the association between cumulative ACEs and adolescent school outcomes across 24 US‐based studies. As observed in other research on ACEs and adolescence [9, 57], interest in this topic is growing, reflected in the recency of many of our included studies. Overall, increased childhood adversity was significantly associated with negative school outcomes including poorer academic performance (e.g., lower grades, lower standardized test scores), poorer school engagement/connectedness, absenteeism, conduct problems at school, school suspension or expulsion, and ultimately, high school dropout. Further, articles in this review found the classic dose–response pattern between ACEs and negative outcomes, consistent with prior ACE research. Yet, several gaps remain, underscoring the need for further research to clarify which academic outcomes are most strongly associated with cumulative ACE exposure, the mechanisms underlying these associations, and potential protective factors.

### 4.1 Measurement/Operationalization

Although most studies were guided by the ACE framework and used criteria aligning with traditional and expanded ACEs, notable variability remained in the types of included ACEs and in the measurement of specific exposures. This variability is unsurprising given that many studies relied on secondary data, a limitation frequently noted in ACE research [58]. The absence of a standardized definition and uniform assessment methods poses challenges for cross‐study comparability and policy translation. Although cumulative ACE scores are often treated as conceptually equivalent across studies, the meaning of a given ACE count may differ substantially depending on which adversities are included, how they are assessed, and the developmental period captured. For example, a cumulative score that includes only maltreatment and household dysfunction is not directly comparable to one that also incorporates poverty, discrimination, and neighborhood violence. Similarly, ACEs identified through administrative CPS records may capture more severe or system‐involved adversity, whereas youth or caregiver reports may capture experiences that never come to the attention of formal systems. These differences complicate efforts to identify thresholds for risk, compare findings across populations, or translate evidence into screening, prevention, and intervention policies. Future research would benefit from more standardized and transparent ACE measurement approaches. A standardized core set of ACE indicators, supplemented by modular expanded ACE domains relevant to structural and community‐level adversity, could improve comparability while preserving sensitivity to contemporary understandings of childhood adversity.

Measurement of school outcomes also varied considerably, particularly regarding more proximal school processes that influence distal school outcomes. More recent literature has focused on factors preceding high school completion (compared to earlier literature that was more narrowly focused on completion), an important evolution for the development of timely, tailored interventions aimed at preventing dropout. However, the evidence for each distinct precursor (e.g., academic goal setting vs. school suspension) remains limited, requiring continued investigation.

### 4.2 Mediating and Moderating Factors

Several studies examined the potential mediating pathways through which cumulative ACEs influence school outcomes. As illustrated in Figure 2 and aligned with the biopsychosocial model, findings from this review suggest ACEs may influence school outcomes through a sequence of multiple, interconnected physiological, behavioral health, and school‐based pathways. Evidence suggests repeated or chronic adversity may lead to toxic stress, which may then disrupt executive functioning, emotion regulation, and other learning processes. Such processes are then associated with behavioral health, school engagement, school attendance, academic achievement, classroom functioning, relationships with teachers and peers, and other factors. These proximal school experiences are then associated with high school completion.

Current findings regarding the mediational effects of behavioral health, particularly the contrasting findings for internalizing and externalizing symptoms warrant closer examination. Externalizing behaviors (e.g., impulsivity, aggression, conduct problems) may be especially likely to affect educational attainment because they are highly visible in school settings and may elicit exclusionary discipline, strained teacher‐student relationships, or disengagement from school. In contrast, internalizing symptoms, particularly depression, may be less visible to educators, manifesting through withdrawal, reduced motivation, concentration difficulties, or absenteeism. Thus, internalizing symptoms may undermine academic functioning through different pathways than externalizing symptoms, leading to differential risks for high school disengagement. The finding that internalizing symptoms were associated with reduced odds of dropout in one study should be interpreted cautiously, as another study found a significant association between internalizing symptoms and dropout, while another study found that depression but not anxiety was associated with dropout.

Future studies should continue testing the pathways from ACEs, distinct behavioral health symptoms, school engagement, disciplinary experiences, academic performance, and educational attainment using longitudinal designs. Studies should also consider whether these mechanisms differ by ACE subtype or developmental timing, school climate, or demographic characteristics, as these factors might shape both exposure to adversity and institutional responses to student behavior.

Evidence of moderation was sparse and mixed. One study found school connectedness modestly buffered ACE‐related risks for absenteeism and achievement [5], suggesting supportive school relationships may mitigate some consequences of adversity. One study found school suspension predicted dropout more strongly for Black youth exposed to ACEs versus White youth [41]; however, a different study found no variation in the association between ACEs and suspension/expulsion by demographic characteristics [46]. Together, these findings suggest that moderation in the ACE–school outcome association may be outcome‐specific and context‐dependent. Future research should examine individual, relational, school, and structural factors that may buffer or exacerbate ACE‐related educational risk.

### 4.3 Implications for School Health, Policy, Practice, and Equity

This review finds that youth with cumulative ACEs are at substantial risk of experiencing negative school outcomes across a variety of domains and, therefore, interventions aimed at mitigating such negative effects for this population are urgently needed. However, additional research on this complex relationship, specifically on the mediating pathways, potential protective factors, and identification of subpopulations most at risk, is a critical first step in developing effective interventions. Preliminary findings in this review indicate behavioral health conditions and factors like school engagement, absenteeism, and academic achievement might act as mediators leading to high school completion, suggesting potential points of intervention. Further, while this review primarily focuses on the downstream effects of cumulative ACE exposure on academic outcomes, we concurrently emphasize the importance of upstream, primary prevention of ACE exposures. Efforts to develop individual‐level interventions are critical for addressing the negative effects of ACEs, but continued attention on community‐ and policy‐level interventions aimed at preventing ACEs is necessary for the long‐term promotion of population wellbeing.

### 4.4 Limitations and Strengths

This review and the included studies have limitations. Although samples represented diverse subpopulations—including groups traditionally underrepresented in research and those at heightened risk for ACE exposure—academic outcomes varied considerably. Continued research examining these relationships, with attention to demographic differences, is essential. This review focused exclusively on US‐based samples and excluded gray literature and non‐English publications, which may limit generalizability to other cultural contexts. Social desirability and reporting bias are possible given the sensitivity of ACE items, though some studies supplemented self‐reports with CPS records. Strengths include a comprehensive search strategy developed with an informationist and screening conducted by two independent reviewers, with audits to ensure fidelity. High interrater reliability suggests minimal reviewer bias. Moreover, most included studies provided robust evidence.

## 5 Conclusion

Although ACEs are well‐established predictors of various negative outcomes, this review underscores their proximal influence during adolescence, where ACEs are associated with poorer academic outcomes. Specifically, cumulative ACEs demonstrated significant negative associations with high school graduation and proximal indicators, including engagement, attendance, and achievement. Further research should explore the mechanisms linking ACEs to academic outcomes, identify resilience‐promoting factors, and advance multi‐level prevention strategies aimed at improving educational attainment and fostering youth wellbeing.

## Funding

This work was made possible through a grant from the University of Michigan School of Nursing from a generous gift from Donald and Karin Allen. This work was completed while C. Polick was supported by the National Institutes of Health/National Institute of Nursing Research grant #T32NR016914 Complexity: Innovations to Promote Health and Safety. Similarly, C. Moore was supported by the National Institute of Nursing Research of the National Institutes of Health under Award Number F31NR020838, by the National Institutes of Health/National Institute of Nursing Research Predoctoral Fellowship Training Grant (T32 NR016914 Complexity: Innovations in Promoting Health through Team Science; Program Director: McCullagh), and by the University of Michigan School of Nursing during completion of this work. The content is solely the responsibility of the authors and does not necessarily represent the official views of the National Institutes of Health or the other funders.

## Conflicts of Interest

The authors declare no conflicts of interest.

## Supporting information


**Table S1:** Specific school variables assessed in included studies. *, significant outcome; M, mediator (partially); mod, moderator.

## Data Availability

Data sharing not applicable to this article as no datasets were generated or analysed during the current study.
