# Supplementary material for: Cumulative Adverse Childhood Experiences (ACEs) and School Outcomes During Adolescence: A Scoping Review
Source: J Sch Health. 2026 Aug 10;96(9):e70209. doi: 10.1111/josh.70209 (PMC13457977; doi:10.1111/josh.70209)
Supplement: Supplementary file 1 [file JOSH-96-0-s001.docx]

**Supplemental Table 1**. Specific school variables assessed in included studies. * = significant outcome; M = mediator (partially); mod = moderator

| **First Author, Year** | **High School Completion** | **Academic Performance/ Achievement** | **School Engagement/ Connectedness** | **Attendance** | **Suspension/**  **Expulsion** | **Grade Repetition** | **Problems Reported to Parents** | **Academic Goal Setting** | **Fighting** | **Growth Mindset of Intelligence** | **Perceived Safety** | **Combination Measures** |
| --- | --- | --- | --- | --- | --- | --- | --- | --- | --- | --- | --- | --- |
| **Cross sectional studies (n=13)** | | | | | | | | | | | | |
| Augustinović, 2026 |  |  | * |  |  |  |  |  |  |  |  |  |
| Boccio, 2025 |  | M | M | * | M |  |  |  |  |  |  |  |
| Crouch, 2019 |  |  | * | * |  | * |  |  |  |  |  |  |
| Duke, 2020 |  | * | mod | * |  |  |  |  |  |  |  |  |
| Ghaffari, 2024 |  | * |  |  |  |  |  |  |  |  |  |  |
| Gussin, 2025 |  |  | * | * |  |  |  |  |  |  |  |  |
| Hartwell, 2024 |  | * | * |  |  |  | * |  |  |  |  | Latent Variable |
| Johnson R., 2021 |  | * |  |  |  |  |  |  | * |  |  |  |
| Kernan, 2025 |  |  |  |  | * |  |  |  |  |  |  |  |
| Lurie, 2023 |  | * |  |  |  |  |  |  |  | M |  |  |
| Moore, 2025 | * |  |  |  |  |  |  |  |  |  |  |  |
| Suleiman, 2021 |  |  | * | * |  |  | * |  |  |  |  | Index of other 3 outcomes |
| Tsevat, 2025 |  |  |  | * |  |  |  |  |  |  |  |  |
| **Longitudinal studies (n=11)** | | | | | | | | | | | |  |
| Brumley, 2021 |  |  |  |  |  |  |  | NS |  |  |  |  |
| Giovanelli, 2016 | * |  |  |  |  |  |  |  |  |  |  |  |
| Giovanelli, 2020 | * |  |  |  |  |  |  |  |  |  |  |  |
| Kallman, 2025 |  | * |  |  |  |  |  |  |  |  |  |  |
| Johnson M, 2018 | * |  |  |  |  |  |  |  |  |  |  |  |
| Leban, 2021 | * |  |  |  | mod |  |  |  |  |  |  |  |
| Lui, 2023 | * |  |  |  |  |  |  |  |  |  |  |  |
| Morrow, 2018 | * | * / M |  |  |  |  |  |  |  |  |  |  |
| Mullins, 2021 |  | * | NS |  |  |  |  |  |  |  |  |  |
| Pan, 2020 | * |  |  |  |  |  |  |  |  |  |  |  |
| Pierce, 2022 |  |  |  |  | * |  |  |  |  |  |  |  |
